# The E3 Ligase AtRDUF1 Positively Regulates Salt Stress Responses in *Arabidopsis thaliana*

**DOI:** 10.1371/journal.pone.0071078

**Published:** 2013-08-12

**Authors:** Junhua Li, Yingying Han, Qingzhen Zhao, Chunhua Li, Qi Xie, Kang Chong, Yunyuan Xu

**Affiliations:** 1 Key Laboratory of Plant Molecular Physiology, Institute of Botany, Chinese Academy of Sciences, Beijing, China; 2 College of Life Sciences, Henan Normal University, Xinxiang, Henan, China; 3 Institute of Genetics and Developmental Biology, Chinese Academy of Sciences, Beijing, China; 4 Key Laboratory of Molecular Biology, College of Life Sciences, Heilongjiang University, Harbin, Heilongjiang, China; Instituto de Biología Molecular y Celular de Plantas, Spain

## Abstract

Ubiquitination is an important post-translational protein modification that is known to play critical roles in diverse biological processes in eukaryotes. The RING E3 ligases function in ubiquitination pathways, and are involved in a large diversity of physiological processes in higher plants. The RING domain-containing E3 ligase AtRDUF1 was previously identified as a positive regulator of ABA-mediated dehydration stress response in *Arabidopsis*. In this study, we report that AtRDUF1 is involved in plant responses to salt stress. *AtRDUF1* expression is upregulated by salt treatment. Overexpression of *AtRDUF1* in *Arabidopsis* results in an insensitivity to salt and osmotic stresses during germination and seedling growth. A double knock-out mutant of *AtRDUF1* and its close homolog *AtRDUF2* (*atrduf1atrduf2*) was hypersensitive to salt treatment. The expression levels of the stress-response genes *RD29B*, *RD22*, and *KIN1* are more sensitive to salt treatment in *AtRDUF1* overexpression plants. In summary, our data show that *AtRDUF1* positively regulates responses to salt stress in *Arabidopsis*.

## Introduction

Ubiquitination is a mechanism of post-translational regulation. The ubiquitination cascade is catalyzed by ubiquitin-activating enzyme (E1), ubuiquitin-conjugating enzyme (E2) and ubiquitin protein ligase (E3). There are >1300 predicted E3 ligases in the *Arabidopsis* genome, including >450 RING type E3s [1], [2]. The vast majority of E3 uibiquitin ligases in the *Arabidopsis* genome facilitate the identification of specific substrates and their subsequent ubiquitination [1], [3]. The E3 ubiquitin ligases are a huge and varied family of proteins and protein complexes which contain either a HECT domain or a U-box/RING domain. The HECT domain subfamily of E3s is relatively small in *Arabidopsis*. The RING domain subfamily of E3s is large in *Arabidopsis* and can be further divided into single subunit RING E3s, such as Constitutive Photomorphogenesis1 (COP1) [4], SEVEN IN ABSENTIA IN ARABIDOPSIS THALIANA 5 (SINAT5) [5], and Arm Repeat-Containing 1 (ARC1) [6], and multisubunit RING E3s including the SCF, CUL3-BTB, and APC complexes [7]. The RING E3s typically contain a cross-brace structure formed of eight Cys and His residues that coordinates two zinc ions [8], [9], [10]. E3 ligases are involved in various aspects of plant biological processes, including growth, development, and protection from biotic and abiotic stresses [11], [12].

To adapt to stressful conditions such as drought, cold, and salinity, plants have developed redundant and sophisticated response strategies which function throughout their life cycle [13], [14], [15]. Plants subjected to stress often accumulate abscisic acid (ABA), an important phytohormone that can protect plants from damage induced by drought, salinity, and pathogenic attack [16], [17]. The accumulation of compatible osmolytes such as proline under dehydration conditions allow cells to maintain osmotic balance with the extracellular space and help to protect the activities of the enzyme activity [18].

AtRDUF1 and AtRDUF2 are homologous proteins with a domain-of-unknown-function (DUF) 1117 motif in their C-terminal regions. Both proteins were identified as ABA-, salt-, and drought-inducible RING finger domain-containing E3 ligases [19]. A study using knock-out mutations revealed that AtRDUFs are positive regulators of ABA response and drought tolerance [19]. Here, through the use of overexpression and knock-out materials, we show that AtRDUF1 positively participates in the response of plants to salt stress.

## Results

### Characterization of AtRDUF1 protein

In order to identify stress-related genes, we analyzed several publicly available databases of *Arabidopsis* microarray experiments. A gene family with three genes (*At5g59550*, *At3g46620* and *At2g39720*) that encode DUF1117 containing RING finger proteins from stress-specific expression profiles in Genevestigator [20] attracted our interest. The microarray data showed that the expression of these genes was up-regulated by several kinds of abiotic stresses. Of particular interest were the data showed that *At5g59550* and *At3g46620* were up-regulated 5.5 and 2.9-fold, respectively, after salt treatment for 6–24 h. *At5g59550* and *At3g46620* were previously designated as *AtRDUF2* and *AtRDUF1*, respectively [19].

**Figure 1:**
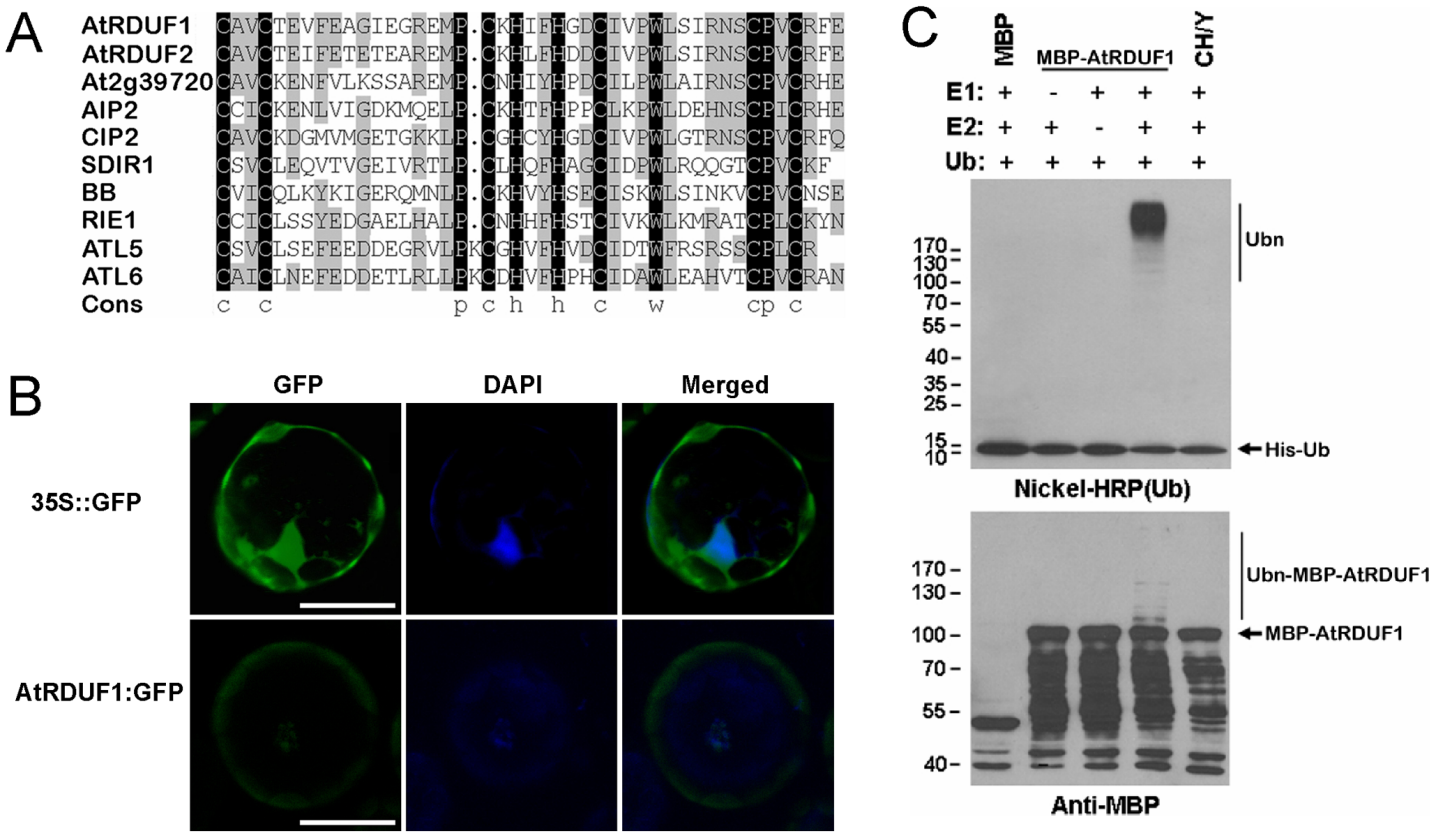
Analysis of the AtRDUF1 protein. (A) Alignment of the RING finger domains of the AtRDUF1 homologs in *Arabidopsis*. Black and gray indicate 100% and ≥50% identities, respectively. (B) Subcellular localization of AtRDUF1:GFP fusion protein in *Arabidopsis* leaf protoplast cells. Bars represent 20 μm. The green and blue fluorescenece are GFP and 4′,6-diamidino-2-phenylindole (DAPI) signals, respectively. (C) Verification of E3 ligase activity of AtRDUF1 by *in vitro* autoubiquitination assay. CH/Y represents the mutant form of the MBP:AtRDUF1 fusion protein, with substitution of metal ligand positions Cys-3, His-4, and His-5 of the RING motif with Tyr. The numbers at left denote the molecular masses of marker proteins in kilodaltons. Nichel-HRP (Ub), the nickel-horseradish peroxidase used to detect His-tagged ubiquitin. Anti-MBP, the anti-MBP antibody to detect maltose fusion proteins.

The AtRDUF1 protein contains a conserved C3H2C3-type RING domain, which shows similarity with many known proteins in *Arabidopsis* (Figure 1A), including several proteins known to be involved in ABA and/or stress signaling pathways, such as ABI3-interacting protein 2 (AIP2) [21], SALT- AND DROUGHT-INDUCED RING FINGER 1 (SDIR1) [22] and ATLs [23], [24]. One of these homologous proteins, RING finger protein for embryogenesis (RIE1), is required for normal development of seeds [25].

Subcellular studies using a 35S::AtRDUF1:GFP fusion protein in *Arabidopsis* leaf protoplast cells showed that the fusion protein was mainly found in the cytosol and in the nuclei (Figure 1B). We examined whether AtRDUF1 is an E3 ligase using *in vitro* methods. As shown in Figure 1C, in the presence of E1 and E2, autoubiquitination of MBP:AtRDUF1 can be detected in the presence of E1 and E2 by both nickel-horseradish peroxidase as well as by anti-MBP antibody assay, indicating that AtRDUF1 is an active E3 ligase. A mutant allele with substitution of metal ligand positions Cys-3, His-4, and His-5 of the RING motif with Tyr (CH/Y) was completely inactive (Figure 1C), indicating that an intact RING motif is essential for the E3 ligase activity of AtRDUF1.

### Expression pattern of *AtRDUF1*

**Figure 2:**
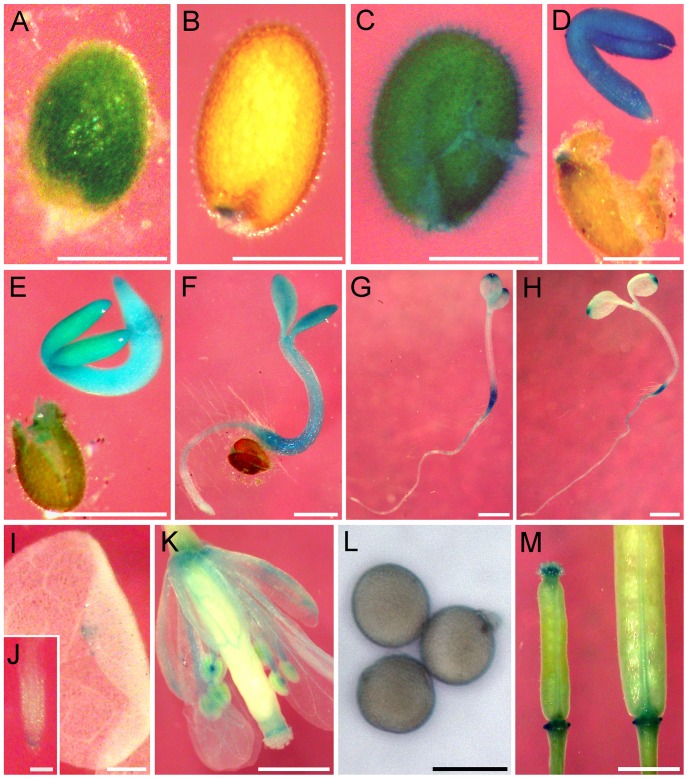
Histochemical localization of GUS activity in *AtRDUF1::GUS* transgenic plants. (A) Developing seed at 12 days after pollination. (B) Desiccated mature seed. (C) Broken mature seed. (D) Dissected seed after imbibition. (E–H) Germinating seedlings at 1-day (E), 2-days (F), 3-days (G), and 4-days (H) after germination. (I) Leaf. (J) Root. (K) Flower. (L) Pollen. (M) Siliques. Bars represent 0.25 mm in A-D; 0.1 mm in J; 10 μm in L; and 1 mm in E-I, K, and M.

To investigate the tissue-specific expression pattern of *AtRDUF1*, a fusion gene comprising the native *AtRDUF1* promoter, a 1.3-kb fragment upstream of the start codon of *AtRDUF1* CDS, and the β-glucuronidase (GUS) gene [26] coding sequence as the reporter gene were constructed and transformed into wild-type *Arabidopsis*. Histochemical staining showed that *AtRDUF1* expressed abundantly in seeds, but was also locally detectable in flowers, hypocotyls, leaves and roots (Figure 2). The staining was strong in immature seeds (Figure 2A), whereas in intact desiccated seeds, the GUS expression was only detectable at the funiculus attachment region (Figure 2B). In broken seeds, GUS staining was uniformly presented throughout the seed (Figure 2C), indicating that the limitation of GUS staining in intact seeds was due to blocking by the seed coat. To exclude possible false observations caused by the diffusion of the soluble intermediate of GUS substrates [27], [28], seeds were dissected and stained separately. The GUS staining signal could be observed uniformly throughout the embryo, but only in the funiculus attachment region of the seed coat (Figure 2D). During germination, a reduction in GUS staining was detectable early on (Figure 2E–H), which is consistent with the decrease of *AtRDUF1* expression detected by real-time qRT-PCR (Figure S2 in File S1). In 4-d-old seedlings, the GUS expression was mainly detected in the junction of the root and hypocotyl, in leaf tips, and around the meristem (Figure 2H). In 2-week old seedlings, the GUS staining was only detectable in leaf tips and root tips (Figure 2I and 2J). In reproductive tissue, *AtRDUF1::GUS* activity was observed at the junction of carpels and pedicels, as well as in stigma, anthers, and pollen, and low levels were detected in the vascular tissues of sepals and petals (Figure 2K–M).

### *AtRDUF1* positively regulates plant salt and osmotic stress responses

To investigate the function of *AtRDUFs*, mutants with T-DNA insertions within the exons of the *AtRDUFs* were obtained and verified (Figure S1A–B in File S1). Expression of the mutant genes was not detected by RT-PCR with primers spanning the T-DNA in the respective homozygous mutants (Figure S1C in File S1). The mutants for *AtRDUF1* and *AtRDUF2* were named *atrduf1-2* and *atrduf2-1*, respectively (hereinafter called “*atrduf1*” and “*atrduf2*”). The double mutant *atrduf1atrduf2* was generated by crossing.

We generated transgenic *Arabidopsis* plants with constitutive expression of *AtRDUF1*, driven by cauliflower mosaic virus (CaMV) 35S promoter. Six transgenic lines of *AtRDUF1* were obtained (Figure S1D and S1E in File S1). The *35S::AtRDUF1* plants showed a wild-type growth phenotype under normal conditions.

**Figure 3:**
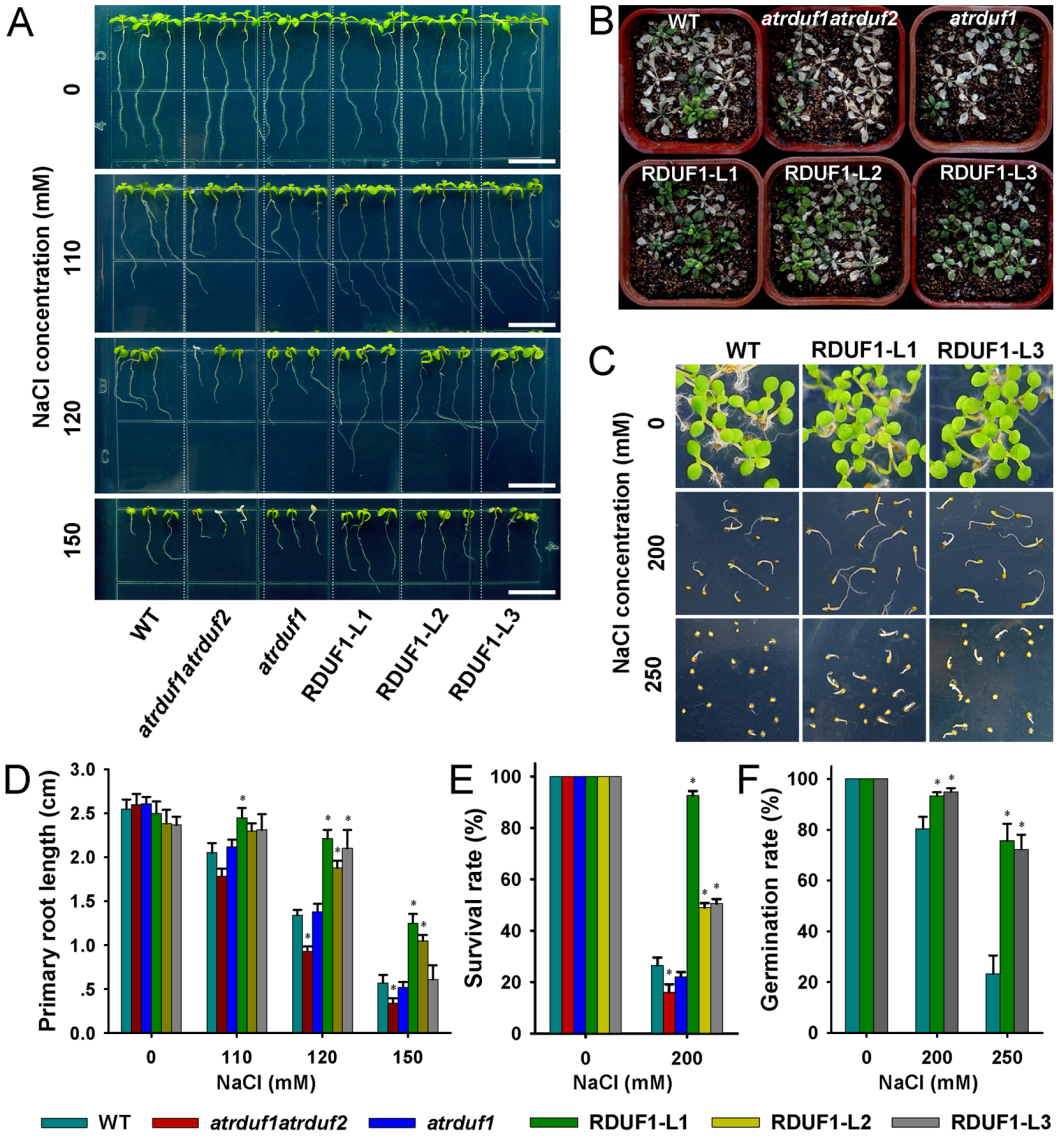
Salt tolerance of *AtRDUF* overexpression plants and mutants. (A) WT, *atrduf1atrduf2*, *atrduf1*, RDUF1-L1 (Line 1 of *35S::AtRDUF1* transgenic plants), RDUF1-L2 and RDUF1-L3 seedlings with or without salt treatment. 3-day-old seedlings were transferred to 1/2 MS medium containing 0, 110, 120 or 150 mM NaCl, and vertically cultured for 6 days. Bars represent 1 cm. (B) Soil-grown plants under salt treatment. 2-week-old soil-grown plants were treated with 200 mM NaCl for 15 days. (C) Germination of seeds at 10 days after imbibition on medium supplemented with 0, 200, or 250 mM NaCl, respectively. (D-F) Primary root length (D), survival rates (E) and germination rates (F) of materials under the conditions described in (A), (B), and (C), respectively. Data are presented as means ± SD. Asterisks indicate significance (*, P<0.05 versus WT control).

The expression of the *AtRDUFs* had been shown to be induced by salt treatment [19]. We therefore tested whether *AtRDUF1* plays a role in plant responses to salt. Seeds of wild-type (WT), *atrduf1atrduf2*, *atrduf1*, and *AtRDUF1* overexpression lines were germinated vertically in 1/2 MS medium. 3-day-old seedlings were transferred to 1/2 MS medium supplemented with 110, 120 or 150 mM NaCl. In the control condition, no significant difference in the length of primary roots was observed among any of the materials (Figure 3A and 3D). Under salt stress conditions, the primary roots of *35S::AtRDUF1* seedlings grew faster than did wild-type seedlings, and the *atrduf1atrduf2* double mutants showed inhibited growth (Figure 3A and 3D).

Germinated *atrduf1atrduf2*, *atrduf1* and *35S::AtRDUF1* lines were transplanted to soil for two weeks, and watered with 200 mM NaCl solution for 15 days to induce salt stress. *35S::AtRDUF1* seedlings exhibited higher survival rates than did WT seedlings. Contrastingly, the survival rate of the salt treated *atrduf1atrduf2* double mutant seedlings was lower than the WT control seedlings (Figure 3B and 3E).

After imbibition on 1/2 MS medium containing NaCl and transfer to a growth chamber for 10 days, the germination rates of *35S::AtRDUF1* seeds were higher than that of WT seeds (Figure 3C and 3F). Therefore, the germination ability of *AtRDUF1* overexpression seeds is insensitive to salt treatment. In our salt tolerance tests, no significant difference was observed between WT and the *atrduf1* mutant.

**Figure 4:**
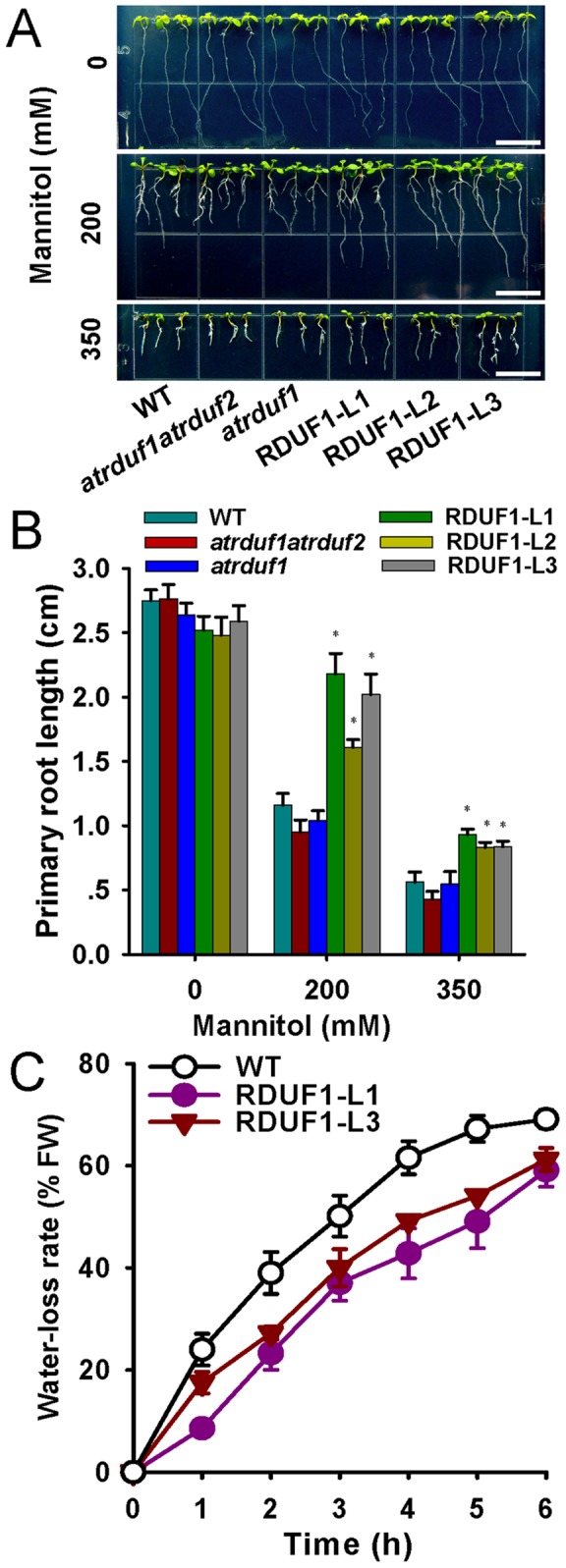
Osmotic tolerance and detached leaf water-loss rates of *AtRDUF1* overexpression plants. (A) WT, *atrduf1atrduf2*, *atrduf1*, RDUF1-L1, RDUF1-L2 and RDUF1-L3 with or without osmotic stress treatment. 3-day-old seedlings were transferred to 1/2 MS medium containing 0, 200, or 350 mM mannitol, and vertically cultured for 6 d. Bars represent 1 cm. (B) Statistical comparison of root lengths of seedlings under the conditions described in (A). (C) Water loss rates of detached leaves. Detached rosette leaves from wild-type and *AtRDUF1* overexpression seedlings were incubated for 6 h at room temperature. Water-loss rate is calculated as the ratio between water loss and plant initial fresh weight (FW), expressed in %. Data are presented as means ± SD. Asterisks indicate significance (*, P<0.05 versus WT control).

Salinity causes ionic and osmotic stresses in plant cells. Germinated transgenic seedlings were transferred to MS medium supplemented with mannitol, a nonmetabolizable sugar, which is known to be used as an osmotic agent in some studies [29], [30], [31]. The root growth of *35S::AtRDUF1* seedlings was less severely inhibited by mannitol than that of WT seedlings (Figure 4A and 4B). Therefore, the tolerance of *35S::AtRDUF1* seedlings to salt treatment is at least partly osmotic in nature.

In contrast with the accelerated water loss in detached leaves of the atrduf1 mutant [19], the water loss of rosette leaves in the overexpression lines was slower than that of WT leaves (Figure 4C). The overexpression plants also showed a sensitive response to ABA in terms of primary root length (Figure S3 in File S1). Our results support that AtRDUF1 positively participates in ABA-mediated dehydration stress responses.

**Figure 5:**
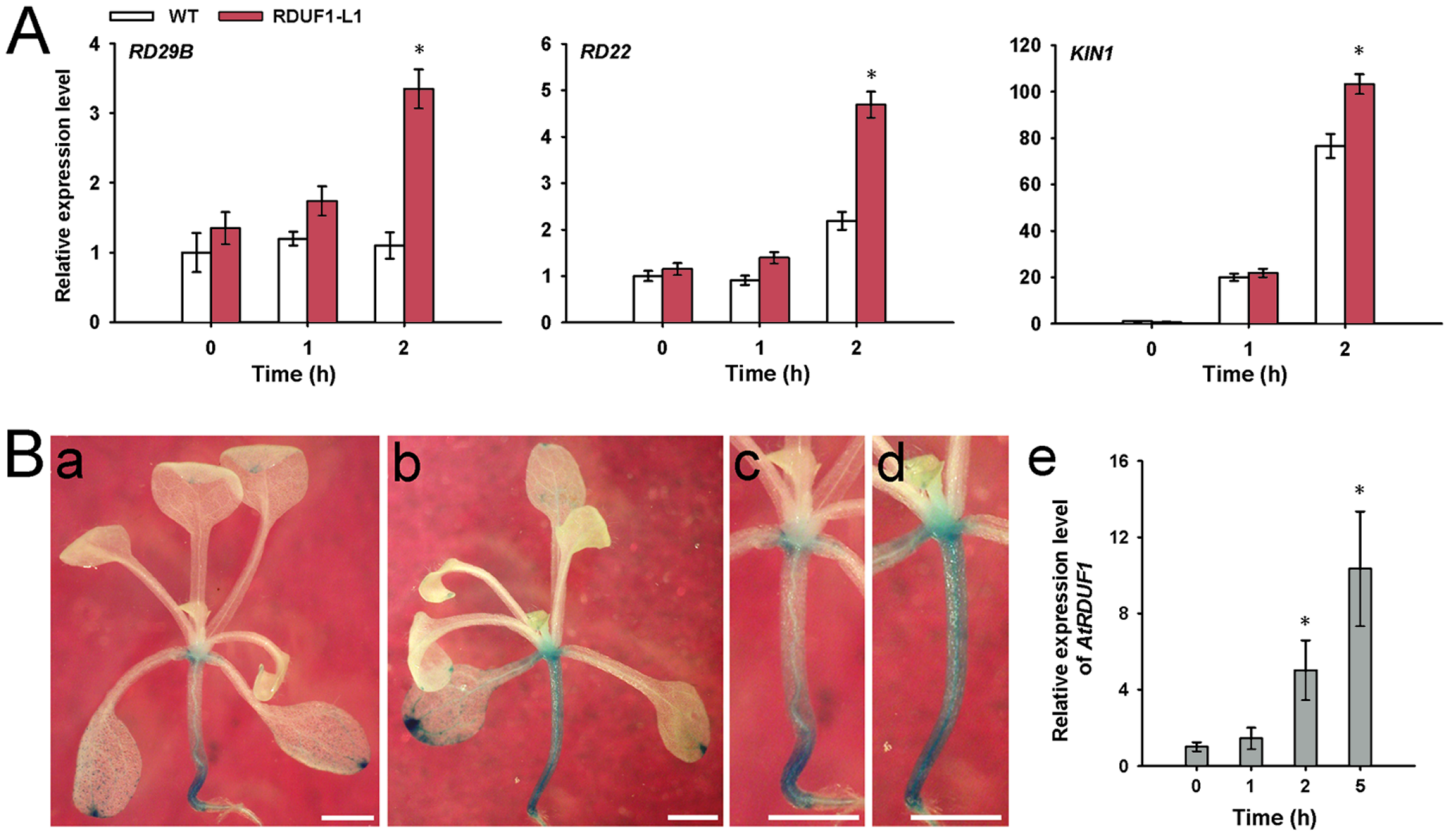
Induction studies of salt-responsive genes and *AtRDUF1*. (A) Induction profiles of salt-responsive genes in wild-type and *AtRDUF1* overexpression plants under salt stress. Transcript levels of *RD29B*, *RD22*, and *KIN1* were determined by real-time qRT-PCR analysis of seedlings treated with 200 mM NaCl. Data represent means ± SD. Mean values were normalized to the transcript levels of an internal control TUBULIN. Asterisks indicate significance (*, P<0.05 versus WT control). (B) Induction of AtRDUF1 expression by salt treatment. Compared with the mock treated plants (a and c), GUS expression was enhanced in *AtRDUF1::GUS* plants treated with 300 mM NaCl for 5 h (b and d). (c) and (d) are close-up views of partial regions of (a) and (b), respectively. Transcript expression values of *AtRDUF1* were also determined by real-time qRT-PCR in 2-week-old plants treated with 300 mM NaCl (e). Bars represent 1 mm. Data represent means ± SD. Mean values were normalized to the transcript levels of an internal control TUBULIN. Asterisks indicate significance (*, P<0.05 versus 0 h control).

We investigated the expression profiles of several stress-responsive genes in the *AtRDUF1* overexpression lines grown under salt stress conditions. 10-day-old seedlings grown on 1/2 MS agar plates were sprayed with 200 mM NaCl solution, the seedlings were harvested after 1 h and 2 h, for extraction of total RNA. The result showed that in RDUF1-L1 seedlings, the transcription levels of *RD29B*, *RD22*, and *KIN1* increased more than those of the WT after 2 h of salt treatment (Figure 5A), suggesting that AtRDUF1 may directly or indirectly interact with known abiotic stress response pathways. When the *AtRDUF1::GUS* transgenic plants were subjected to salt treatment, the GUS staining was enhanced compared with control plants (Figure 5Ba–5Bd), which is consistent with the real-time qRT-PCR results (Figure 5Be).

## Discussion

In the course of our investigation of likely stress-related genes, we identified *AtRDUFs* from *in silico* data. Their putative proteins contain both RING finger and DUF1117 domains, and represent a novel E3 ligase family in plants. Real time qRT-PCR and promoter-GUS analyses confirmed that the transcription of *AtRDUF1* was indeed salt inducible (Figure 5B). We analyzed the phenotypes of wild-type as well as overexpression and loss-of-function mutants of *AtRDUFs* following salt treatment. Our results showed that *AtRDUF1* positively regulates plant responses to salt treatment during both germination and post-germination growth (Figure 3). Furthermore, *AtRDUF1* also positively regulates plant tolerance to osmotic and dehydration stress (Figure 4).

The fluorescence of AtRDUF1:GFP fusion protein was detectable in our subcellular localization study (Figure 1B), and *35S::AtRDUF1* transgenic plants showed obvious tolerance to salt, osmotic, and water loss stresses (Figure 3 and 4). In a similar report, a fusion protein approach was used to develop transgenic plants that overexpressed AtRDUF1. However, the authors were unsuccessful in detecting significant accumulation of the fusion protein, in spite of expression of significant amounts of *AtRDUF1* mRNA, so the plants were not extensively analyzed [19]. The discrepancy of detection may be attributable to distinctions in vector efficiency or differences in the sensitivity of the assay methods employed in these independent studies.

In addition to the results reported here, *AtRDUF1* and *AtRDUF2* are also known to be up-regulated by chitin treatment [32]. Among the homologous proteins of the AtRDUFs subfamily, the E3 ligases KEG [12] and AIP2 [21] are negative regulators of ABA signaling, acting by targeting and degrading ABI5 and ABI3, respectively. The E3 ligase SDIR1 positively regulates ABA signaling, and the *sdir1* mutant is resistant to salt and drought stress [22]. Therefore, it is apparent that AtRDUFs and their homologous proteins are widely involved in plant adaptations to stress.

We were able to confirm that AtRDUF1 has E3 ligase activity by ubiquitination assays (Figure 1C). Ubiquitination has been shown to play an important role in the perception and transduction of various internal and external environmental signals [33], [34]. To test whether AtRDUF1 affects the expression of known stress pathway genes, several marker genes in the stress-responsive pathways were analyzed, *RD29B*, *RD22*, and *KIN1* showed a hypersensitive salt response in transgenic plants (Figure 5A). *RD29B* is a cold-, high salt-, and dessication-inducible gene with two ABA-responsive elements (ABREs) present in its promoter region [35]. *RD22* transcription is induced by salt and ABA treatment, but no ABRE was identified in its promoter region [22], [36]. *KIN1*, which contains the C repeat/dehydration-responsive element (CRT/DRE) motif in its promoter, can be induced by cold, ABA and dehydration treatment [37]. According to our data and published results [19], AtRDUF1 may be involved in the up-regulation of stress responses in *Arabidopsis* seedlings, in this respect, AtRDUF1, may be similar to SDIR1 and AtSAP5, both of which have been shown to be E3 ligases and are known in promoting stress gene expression and stress tolerance [22], [38], [39].

Glycerol, a compatible osmolyte, is used in defending against dehydration stresses in yeast, marine algae, insects, and amphibians [40], [41], [42], [43]. Accumulation of intracellular glycerol was observed during the salt adaptation processes of many microorganism such as *Aspergillus nidulans* [44] and *Aureobasidium pullulans* [45]. In *Arabidopsis*, the results from studies in mutants with defects in storage lipid accumulation prior to seed maturation or lipid catabolism following germination [29], [30], [31] suggest that glycerol or glycerol-derived lipids could serve as compatible osmolytes in dehydration stress conditions in plants. As *AtRDUF1* expressed primarily in embryos of matured seeds (Figure 2), we tested the correlation of *AtRDUF1* expression with storage lipids (Text S1 in File S1). In young seedlings, the staining of GUS driven by the *AtRDUF1* promoter partially coincided with staining of Sudan red 7B, a fat-soluble dye that stains lipids red, and the Sudan red 7B staining was darker in the *35S::RDUF1* seedlings (Figure S4A in File S1). The levels of triacylglycerol (TAG), the primary seed oil in *Arabidopsis* were higher in seeds and young seedlings of *35S::AtRDUF1* lines than in WT seedlings (Figure S4B in File S1). The vegetative tissues of *35S::RDUF1* seedlings contained a higher content of triglycerides (the ester of glycerol) than did WT control tissues (Figure S4C in File S1). Finally, the oleosin maker gene *OleS3* and triglycerides content change in response to salt stress (Figure S4D and S4E in File S1). We speculate that a mechanism by which AtRDUF1 increases salt tolerance is through the positively regulation of either the delayed catabolism or the increased accumulation of the storage lipids.

In conclusion, our data show that AtRDUF1 is a functional E3 ligase and a positive regulator of the *Arabidopsis* response to salt stress. This study contributes to our understanding of the molecular factors involved in the responses of plants to abiotic stresses.

## Materials and Methods

### Plant materials

All *Arabidopsis* plants used in this study were of the *Columbia* (Col-0) ecotype. T-DNA insertion lines SALK_131634 (for *AtRDUF1*) and N471914 (for *AtRDUF2*) were obtained from ABRC [46] and NASC [47], respectively. Seedlings were grown under long-day conditions (16 h light/8 h dark) at 22°C, 40 to 60% RH and 63 mE·s^−1^·m^−2^ light intensity.

### Vector construction and *Arabidopsis* transformation

The cDNA of *AtRDUF1* was amplified and cloned into the pSN1301 expression vector [48] driven by the CaMV 35S promoter. The primers used for *AtRDUF1* overexpression were R1BamHIF and R1KpnIR (all primer sequences used in this study are listed in Table S1 in File S1). The 1.3 kb promoter sequence of *AtRDUF1* was amplified with primers R1PKpnIF and R1PBamHIR, and cloned into the pGUS1301 vector [49]. The constructed plasmid was introduced into *Agrobacterium tumefaciens* strain C58. *Arabidopsis* was transformed using the floral dip method [50]. Transgenic plants were first screened on medium containing 40 mg/l hygromycin and subsequently transferred to soil. To produce a *AtRDUF1:GFP* fusion gene driven by 35S promoter, the *AtRDUF1* CDS sequence with the stop codon deleted was amplified with primers R1GXhoIF and R1GKpnIR, and cloned in frame into pBI121GFP. To generate the MBP:AtRDUF1 fusion, the *AtRDUF1* CDS was cloned in frame into pMAL-c2 (NEB, Berverly, MA, USA) with primers R1MBamHIF and R1MSalIR. A mutant allele with substitution of metal ligand positions Cys-3, His-4, and His-5 of the RING motif with Tyr (CH/Y) was introduced with primers R1mF and R1mR. *Dpn*I mediated site-directed mutagenesis in the plasmid was performed as described previously [51].

### Subcellular localization

Plasmid 35S::AtRDUF1:GFP and 35S::GFP were purified by the use of Tiangen kits according to the manufacturer's protocols. *Arabidopsis* protoplasts transformations were performed as described previously [52]. For detection of nuclei, samples were stained with DAPI at a final concentration of 1 μg/mL.

### E3 ubiquitin ligase activity assay

The MBP:AtRDUF1 fusion protein was expressed in *Escherichia coli* strain BL21 (DE3), and subsequently purified using amylose resin (NEB). The *in vitro* E3 ubiquitin ligase activity assay was performed as described previously [5]. Following the assay reactions, proteins were separated by SDS-PAGE, blotted, and probed by either HisDetector Nickel-HRP (KPL company, USA) for the detection of His-tagged ubiquitin or antibody to MBP (antiserum; NEB) for detection of the MBP-tagged AtRDUF1 protein. Results were visualized using chemiluminescence as per the instructions of the manufacturer (ECL; Amersham Pharmacia, Amersham, UK).

### Stress and ABA treatment

After surface sterilization, seeds were imbibed at 4°C for 3 d in the dark. The seeds were sown on half-strength Murashige and Skoog medium [53] supplemented with 1% sucrose and 0.7% agar. 3-day-old seedlings were transferred to 1/2 MS medium containing NaCl, mannitol, or ABA. Primary root length was measured after 6 d. 4-day-old seedlings were transplanted to soil and cultured for two weeks, and treated with 200 mM NaCl for 15 days to induce salt stress. For the germination test, all seeds were harvested simultaneously and stored for 5 weeks after harvest. Germination was determined as penetration of the radicle through the seed coat 10 days after imbibition. Each assay was repeated three times. The data in the graphs (Figure 3D-F, 4B–C, S3B, S4C and S4E in File S1) were subjected to analysis of variance (ANOVA) and means were compared by *t*-test at the 5% level. All analyses were performed with version 13.0 of SPSS software.

The water loss assay was performed as described previously [54]. Eight rosette leaves per plant were detached from 3-week-old plants. The leaves were exposed to air and the fresh weights were measured at the indicated time points shown in Figure 4C. Water-loss rate is calculated as the ratio between water loss and plant initial fresh weight, expressed in %.

### GUS histochemical assays

Tissues of transgenic seedlings harboring *AtRDUF1::GUS* at various growth stages were used for the GUS activity assays. Fixation of the tissues by acetone and incubation in staining solution was performed as described by Sieburth and Meyerowitz [55]. For staining of pollen, the pollen was isolated from stamens by vortexing in acetone and enriched using centrifugation at rcf 3000.

### Expression analysis

Total RNA was extracted from tissues with Trizol reagent (Invitrogen, Carlsbad, CA, USA), and treated with RNase-free DNase (Takara, Dalian, China) according to the manufacturer's instructions. 2 μg of total RNA was used for cDNA synthesis with avian myeloblastosis virus (AMV) reverse transcriptase (Promega, Madison, WI, USA) according to the manufacturer's protocol. Real-Time qRT-PCR was performed as described previously [56]. Three independent experiments were performed. The relative quantification method (Delta-Delta CT) was used to evaluate variation in expression. Significance of differences was determined by *χ* ^2^-test. The primer sets for PCR were: R1realtimeF and R1realtimeR for *AtRDUF1*; RD29BF and RD29BR for *RD29B*; RD22F and RD22R for *RD22*; KIN1F and KIN1R for *KIN1*; TubulinF and TubulinR for *TUBULIN*; OleS3F and OleS3R for *OleS3*; ACTINF and ACTINR for *ACTIN*.

## Supporting Information

File S1 **Text S1, lipid detection.** **Figure S1**, verification of T-DNA insertion mutants of *AtRDUFs* and *AtRDUF1* overexpression lines. **Figure S2**, relative quantification of *AtRDUF1* transcription during germination assayed by real-time qRT-PCR. **Figure S3**, response of *AtRDUF1* overexpression plants to ABA. **Figure S4**, effects of AtRDUF1 and salt treatment on plant lipids. **Table S1**, sequences of the oligonucleotides used in this study.
(DOC)
