# Supplementary material for: The E3 Ligase AtRDUF1 Positively Regulates Salt Stress Responses in Arabidopsis thaliana
Source: PLoS One. 2013 Aug 12;8(8):e71078. doi: 10.1371/journal.pone.0071078 (PMC3741333; doi:10.1371/journal.pone.0071078)
Supplement: File S1 — Text S1, lipid detection. Figure S1, verification of T-DNA insertion mutants of AtRDUFs and AtRDUF1 overexpression lines. Figure S2, relative quantification of AtRDUF1 transcription during germination assayed by real-time qRT-PCR. Figure S3, response of AtRDUF1 overexpression plants to ABA. Figure S4, effects of AtRDUF1 and salt treatment on plant lipids. Table S1, sequences of the oligonucleotides used in this study. (DOC) [file pone.0071078.s001.doc]

**Supporting Information**

**Text S1.**

**Lipid detection**

Lipid staining in plant tissue with Sudan Red 7B (Sangon, Shanghai, China) was performed according to previously described methods . To quantify the amount of TAG, total lipids from fifty seeds or seedlings were extracted according to the method described by Bligh and Dyer . One tenth of the final total lipid was used for separation of TAG by thin layer chromatography (TLC). The samples were spotted on a Silica Gel HPTLC plate and developed in hexane:Et_2_O:AcOH (70:30:1, v/v). Triolein was used as a TAG standard. The lipids were visualized by exposing the plate into iodine vapor for 5 min. Triglyceride levels were determined using the Tissue Triglyceride Assay Kit E1013 (Applygen, Beijing, China) according to the manufacturer’s instructions.

**References**

1. Brundrett M, Kendrick B, Peterson C (1991) Efficient lipid staining in pant material with Sudan Red 7B or Fluoral Yellow 088 in polyethylene glycol-glycerol. Biotech Histochem 66: 111-116.

2. Tsukagoshi H, Morikami A, Nakamura K (2007) Two B3 domain transcriptional repressors prevent sugar-inducible expression of seed maturation genes in *Arabidopsis* seedlings. Proc Natl Acad Sci U S A 104: 2543-2547.

3. Blight E, Dyer W (1959) A rapid method of total lipid extraction and purification. Can J Biochem Physiol 37: 911-917.

**
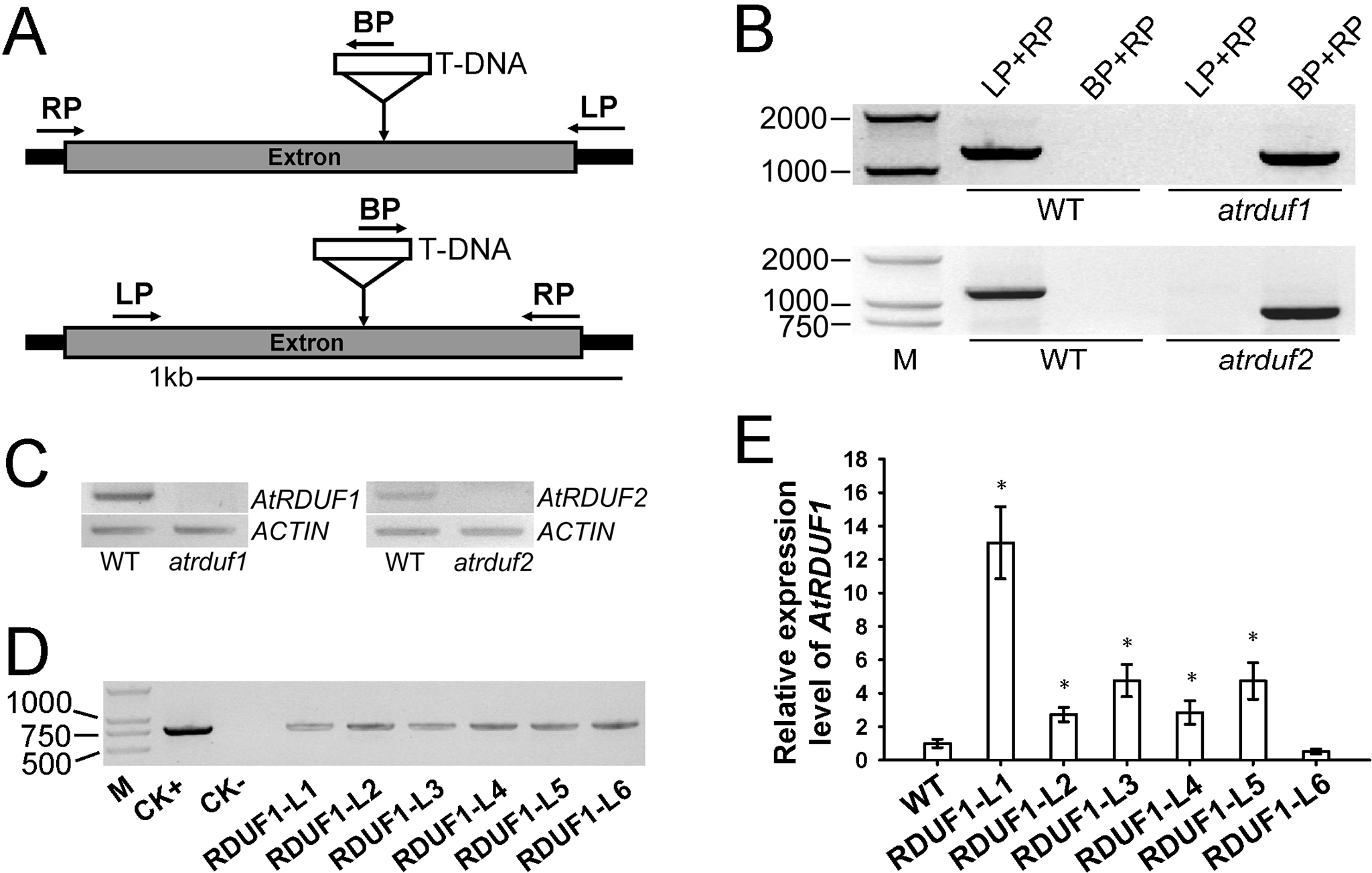
**

**Figure S1. Verification of T-DNA insertion mutants of *AtRDUFs* and *AtRDUF1* overexpression lines.** (A) Illustrations of *AtRDUF* loci, showing the gene organization and the positions of the T-DNA insertion sites and primer sites of *atrduf1* (SALK_131634, upper panel) and *atrduf2* (N471914, bottom panel). Boxes represent exons. LP: left genome primer; RP: right genome primer; BP: T-DNA border primer. (B) Verification of T-DNA insertion. Upper panel, verification of SALK_131634. 131634LP, 131634RP and SALKBP were used as LP, RP and BP primers, respectively. Bottom panel, verification of N471914. N471914LP, N471914RP and pAC161BP were used as LP, RP and BP primers, respectively. M: DNA molecular weight marker. The numbers at left denote the molecular masses of DNA marker in base pairs. (C) Verification of mutants by RT-PCR. Upper panel: detection of *AtRDUF1* mRNA in the homologous mutant SALK_131634 with primer 131634LP and 131634RP. Bottom panel: detection of *AtRDUF2* mRNA in the homologous mutant N471914 with primer N471914LP and N471914RP. (D) Verification of transformation by PCR with *Arabidopsis* genomic DNA as template with primer 35S3'F and R1RTR. CK+: plasmid harbouring *AtRDUF1* as a positive control; CK-: wild-type genomic DNA as a negative control; RDUF1-L1 to RDUF1-L6: genomic DNA from *35S::AtRDUF1* transgenic plant line 1-6, respectively. M: DNA molecular weight marker. The numbers at left denote the molecular masses of DNA marker in base pairs. (E) Relative quantification of *AtRDUF1* transcription by real-time qRT-PCR in *35S::AtRDUF1* transgenic plant lines. Data represent means ± SD. Mean values were normalized to the transcript levels of an internal control *TUBULIN*. Asterisks indicate significance (*, *P*＜0.05 versus WT control).


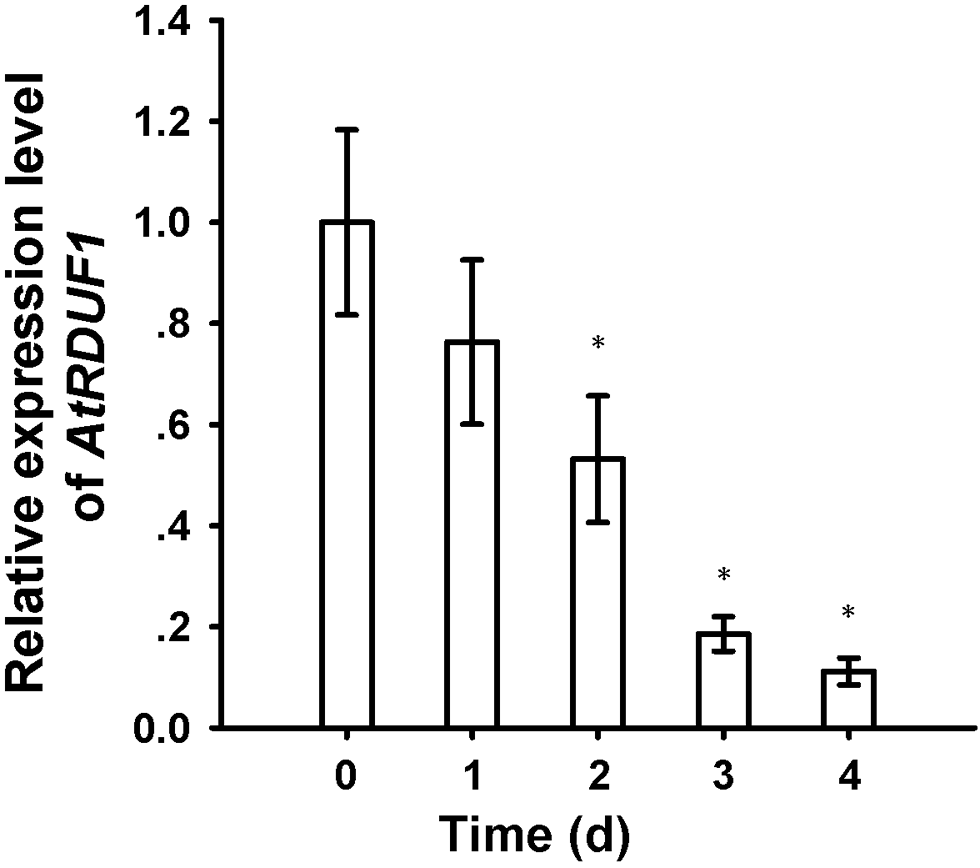


**Figure S2. Relative quantification of *AtRDUF1* transcription during germination assayed by real-time qRT-PCR.** Data represent means ± SD. Mean values were normalized to the transcript levels of an internal control *TUBULIN*. Asterisks indicate significance (*, *P*＜0.05 versus 0 d control).


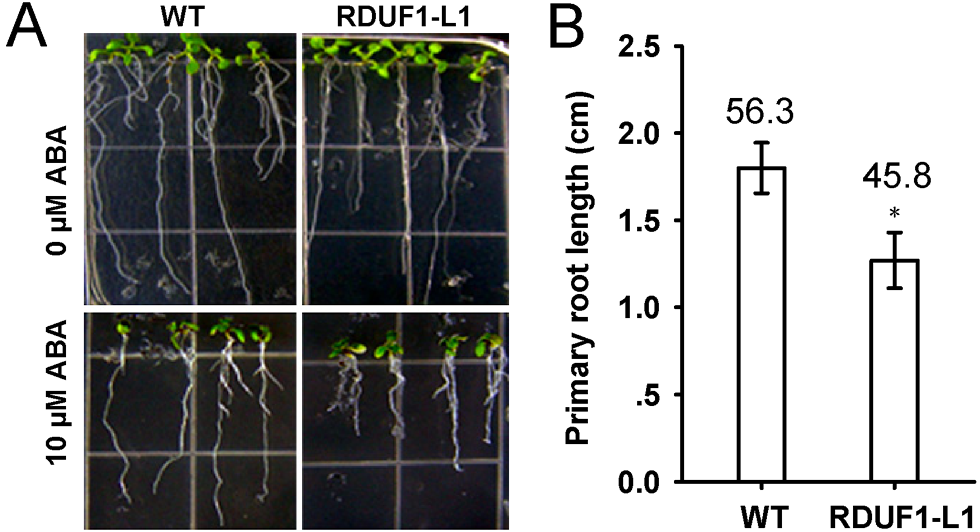


**Figure S3. Response of *AtRDUF1* overexpression plants to ABA.** (A) Wild-type and RDUF1-L1 plants on medium with or without ABA. 3-day-old seedlings of wild-type and RDUF1-L1 plants were transferred to 1/2 MS medium without or with 10 μM ABA and vertically cultured for 6 days. (B) Primary root length of plants treated with 10 μM ABA for 6 days. Values given above the columns are the percentages of the average root length of the ABA treated plants versus untreated control plants. Data are presented as means ± SD. The asterisk indicates significance (*, *P*＜0.05 versus WT control).


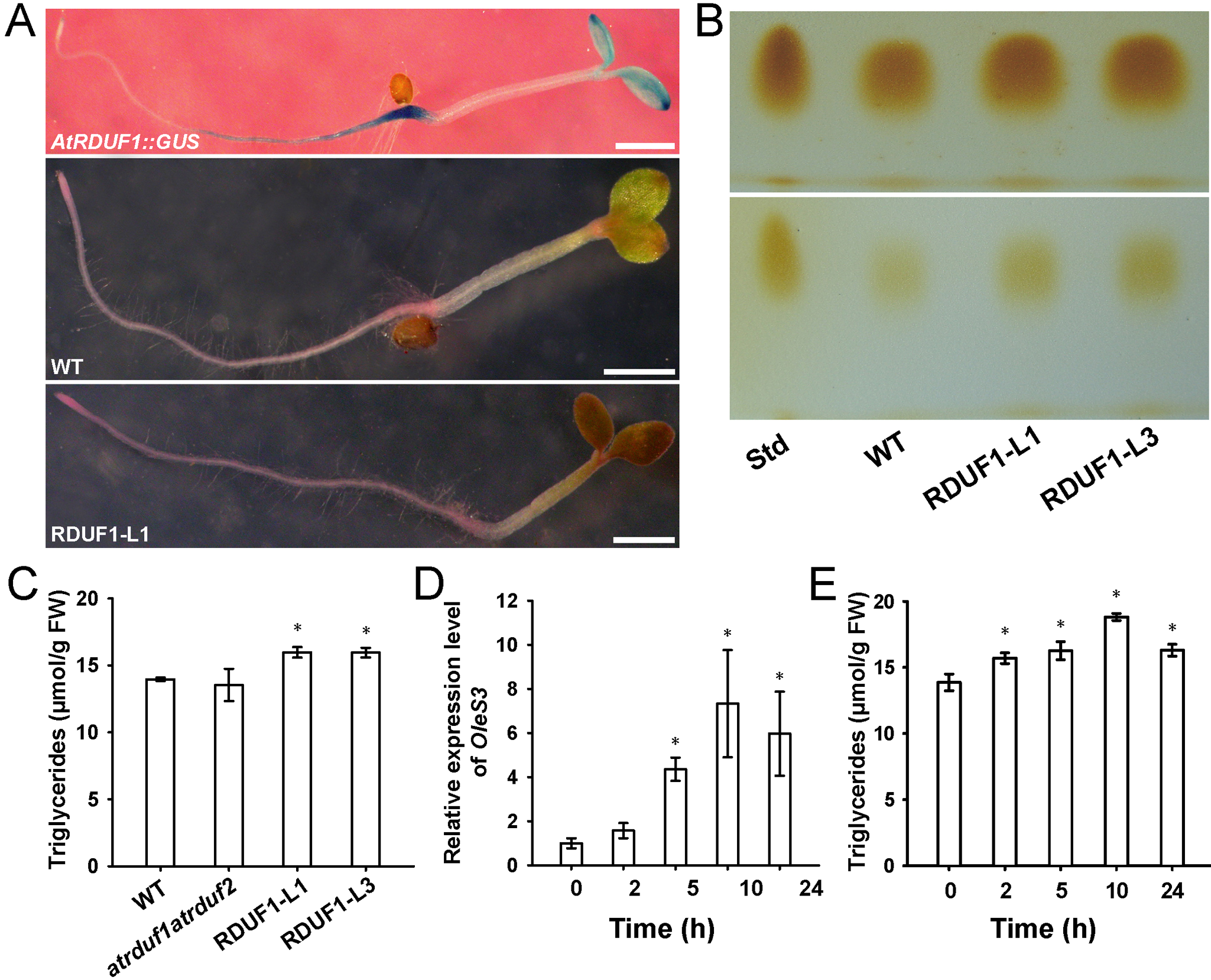


**Figure S4. Effects of AtRDUF1 and salt treatment on plant lipids.** (A) Comparison of the *AtRDUF1* expression pattern (upper panel) and the distribution pattern of neutral lipids stained by Sudan Red in 3-day-old WT (middle panel) and RDUF1-L1 (bottom panel) plants. Bars represent 1 mm. (B) Quantification of triacylglycerol (TAG) separated by thin layer chromatography (TLC), the TAG quantity in the graph is equivalent to that from five seeds (upper panel) or 3-day-old seedlings (bottom panel). (C) Quantification of triglycerides in one-week-old WT, *atrduf1atrduf2*, RDUF1-L1 and RDUF1-L3 seedlings. FW: fresh weight. Data are presented as means ± SD. Asterisks indicates significance (*, *P*＜0.05 versus WT control). (D and E) Induction of *OleS3* expression (D) and triglycerides (E) in one-week-old seedlings treated with 300 mM NaCl. Data are presented as means ± SD. Asterisks indicates significance (*, *P*＜0.05 versus 0 h control).

**Table S1. Sequences of the oligonucleotides used in this study.**

| Name | Sequences (in the 5' to 3' orientation) |
| --- | --- |
| 131634LP | ATCTCACCTAGTTTCATCAGAA |
| 131634RP | TCACCAGCAACACAAGTTAT |
| N471914LP | TGCGACGGCG GTTTTATC |
| N471914RP | TAGAAAACGAAGCACCAATC |
| SALKBP | GCGTGGACCGCTTGCTGCAACT |
| pAC161BP | ATATTGACCATCATACTCATTGC |
| R1BamHIF | CGGGATCCTCACCAGCAACACAAGTTAT |
| R1KpnIR | GGGGTACCATCTCACCTAGTTTCATCAGAA |
| R1GXhoIF | CCTCGAGGATCCGTATGATGCCAAATTCTAGATC |
| R1GKpnIR | GGGGTACCGAAAAATCTTCTGATCAACG |
| R1MBamHIF | CGGATCCATGATGCCAAATTCT |
| R1MSalIR | GCGTCGACTCAGAAAAATCTTCTG |
| R1mF | GAAGGTCGTGAGATGCCGTATAAATACATATTTTACGGTGATTGTATTGTTCCATGGC |
| R1mR | GCCATGGAACAATACAATCACCGTAAAATATGTATTTATACGGCATCTCACGACCTTC |
| R1PKpnIF | GGGGTACCCGGCATCCACAATTCAGGC |
| R1PBamHIR | CGGGATCCCTTTTGAAAATAACTTGTGTTGCTGG |
| 35S3'F | TTCGCAAGACCCTTCCTC |
| R1realtimeF | AGCAACACAAGTTATTTTCAAAAGATG |
| R1realtimeR | TGTGGTGGTTGATTCTGTCGTC |
| RD29BF | AGAAGGAATGGTGGGGAAAG |
| RD29BR | CAACTCACTTCCACCGGAAT |
| KIN1F | CAGACCGCTGGCAAAGCT |
| KIN1R | GGCCTTGTCCAGCAGAACAT |
| RD22F | ATAATCTTTTGACTTTCGATTTTACCG |
| RD22R | CTTGGACGTTGGTACTTTTCTCG |
| TubulinF | GAAACCTTGAAGACAGTCGCAAT |
| TubulinR | GCAATCTGGTGCTGGAAACAA |
| OleS3F | AGGCAGATTGCTAAAGCTGCAAC |
| OleS3R | ACTGTGATGAGAGCCGGG |
| ACTINF | GGTAACATTGTGCTCAGTGGTGG |
| ACTINR | AACGACCTTAATCTTCATGCTGC |

The underlined sequences are the introduced restriction sites.
